# Oxidative stress and endoplasmic reticulum stress contribute to *L. paracasei* subsp. *paracasei* M5L exopolysaccharide‐induced apoptosis in HT‐29 cells

**DOI:** 10.1002/fsn3.2142

**Published:** 2021-01-29

**Authors:** Wei Song, Panpan Hu, Shouli Guo, Jinhong Hu, Chen Song, Tianyi Wang, Zihan Gao, Tianli Yue

**Affiliations:** ^1^ College of Food Science and Technology Northwest University Xi'an China; ^2^ Laboratory of Nutritional and Healthy Food‐Individuation Manufacturing Engineering Xi'an China; ^3^ Research Center of Food Safety Risk Assessment and Control Xi'an China; ^4^ Department of Life Science Luliang University Lv Liang China; ^5^ Animal Experiment Center of the Second Affiliated Hospital Harbin Medical University Harbin China; ^6^ College of Chemical Engineering and Chemistry Harbin Institute of Technology Harbin China; ^7^ National Local Joint Laboratory of Extreme Environmental Nutritional Molecule Synthesis Transformation and Separation Harbin China

**Keywords:** apoptosis, endoplasmic reticulum stress, HT‐29 cells, M5‐EPSs, oxidative stress

## Abstract

Colorectal cancer is the third most malignant cancer occurring around the world. Effective prevention and treatment have been increasingly the focus of global attention. Long‐term diet of fermented dairy inhibits proliferation of colon cancer cell, which is considered that not only live lactic acid bacteria but also the secreted exopolysaccharides exert the function. In this scenario, this study aimed to investigate the mechanism of growth inhibition on HT‐29 cells induced in vitro by exopolysaccharides isolated from *Lactobacillus paracasei* subsp. *paracasei* M5L (M5‐EPSs).

HT‐29 cells which were treated by a set of concentrations of M5‐EPSs have been investigated of cell viability, characteristic changes, cell cycle distribution, and redox system. The results demonstrated that M5‐EPSs treatments induced HT‐29 cell apoptosis and resulted in upregulation of ROS levels and downregulation of antioxidant enzyme activities, leading to an imbalance in the oxidation system in HT‐29 cells. In response to M5‐EPSs, endogenous ER stress (ERS) markers, including GRP78, ATF4, and CHOP, were transcriptionally altered so that activating the ERS in HT‐29 cells. After NAC treatment, the oxidative stress was inhibited, and the expression of GRP78 and CHOP was significantly decreased, indicating that oxidative stress can significantly affect the ERS pathway. Furthermore, it suggested that the occurrence of apoptosis was associated with Bcl‐2 gene family.

In conclusion, this study demonstrated that M5‐EPSs can induce HT‐29 cells apoptosis by destroying the redox system through activation of the ERS signaling pathway.

## Graphical abstract

*Lactobacillus paracasei* subsp. paracasei M5L (M5‐EPSs) induced apoptosis in HT‐29 colon cancer cells associated with regulation of Bcl‐2 gene family; treatments with M5‐EPSs resulted in upregulation of ROS levels and downregulation of antioxidant enzyme activities, leading to an imbalance in the oxidation system in HT‐29 cells; endogenous ER stress (ERS) was involved in HT‐29 cells’ apoptosis; and M5‐EPSs induced HT‐29 cells' apoptosis by destroying the redox system through activation of the ERS signaling pathway.

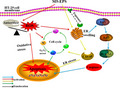

## 1 INTRODUCTION

Colorectal cancer, the third most malignant cancer occurring around the world, is thought to be influenced by many factors, making this form of cancer a major health concern (Bray et al., 2018). Despite Conventional or complementary therapies, including chemotherapy, radiation, surgery, physical rehabilitation, and immunotherapy have been attempted to treat colorectal cancer, a successful treatment has not yet been found, and surgical resection is always used for colorectal cancer treatment (Adam et al.; Delaunoit et al., 2005; Zampino et al., 2016). However, the drug resistance of cancer cells has blocked their apoptosis; in addition, anticancer agents may have cytotoxic effects in normal cells (Alfarouk et al., 2015; Lichan Chen, 2018; Sun et al., 2012). In recent years, an increasing number of natural products with anticancer compounds have had their pharmacological bioactivities confirmed and have been used to explain the mechanisms of cancer prevention in apoptosis.

The endoplasmic reticulum activates the unfolded protein response (UPR) when it undergoes stress. This response can protect cells from the damage caused by the endoplasmic reticulum stress (ERS) and restore cell function; however, when ERS is too strong or lasts too long, the endoplasmic reticulum homeostasis is seriously unbalanced and cannot be repaired, which will lead to cell apoptosis. The UPR normally activates three transcription factors, including inositol‐requiring enzyme 1 (IRE1), PEK‐like endoplasmic reticulum kinase (RERK/PEK), and activating transcription factor 6 (ATF6), which degrade deposited unfolded and misfolded proteins of these three transcription factors, ATF6, as a receptor protein in the endoplasmic reticulum, is one of the factors in the apoptosis and autophagy pathways induced by the ERS (Haque et al., 2015). ERS‐induced death signaling pathways include the CHOP/GADDl53, JNK, and caspase pathways (Wang et al., 2014). Cells enhance ATF4 through the PERK pathway, and CHOP is also a transcription factor of the PERK pathway and the direct target of ATF4. CHOP and caspase expression are weak when homeostasis is balanced. When ERS occurs, CHOP and caspase expression will significantly increase. Overexpression of CHOP and caspase can promote cell cycle stagnation or lead to apoptosis (Liu et al., 2015).

Another pathway that causes cell apoptosis is the oxidative stress pathway (Xiang et al., 2015). Including cancer, inflammation, diabetes, Parkinson's disease, Alzheimer's disease, atherosclerosis, and aging, various disorders and diseases have been considered to be related with massive production of reactive oxygen species (ROS) and oxygen‐derived free radicals. Besides, dysfunction of cells, cell cycle arrest, and apoptosis were also involved in oxidative stress. Safety of synthetic antioxidants has recently been questioned, even though they have been shown to be fairly effective at the processes of slowing down the oxidation (Lu et al., 2017; Moloney & Cotter, 2018; Yuan et al., 2019). Furthermore, severe side effects as well as increased tolerance to the treatments of cancer have been brought by constant use of medical treatment over time. As a result, exploring and utilizing natural antioxidants and cancer prevention agents including polysaccharides have been paying more attention for their lower cytotoxicity (Chen et al., 2019).

Bacterial exopolysaccharides (EPSs) have gradually attracted more attention from researchers because of their structural diversity as well as their versatile physicochemical and biological properties. Among all beneficial bacteria, lactic acid bacteria (LAB) represent some of the best producers of EPSs due to their long history of safe use in food products (Durlu‐Ozkaya et al., 2007; Wu et al., 2010). Much evidence demonstrates that EPSs from LAB exhibit various biological activities, such as probiotic functionality, blood cholesterol‐lowering effects, antioxidation, anticancer activity, and immunomodulation (Cheung et al., 2012; Huang et al., 2007; Wang, Li, et al., 2014).

*Lactobacillus paracasei* subsp. *paracasei* M5L was identified and obtained from our lab. According to our previous research, the cell wall and genomic DNA of *L. paracasei* M5L and even active or inactive strains display biological activity (Hu et al., 2015). Nevertheless, M5‐EPSs mediate the effects in HT‐29 cells by the mechanism, which has not yet been fully examined. Therefore, this study was focusing on the antitumor effects, as well as the apoptotic effects of M5‐EPSs on HT‐29 cells with disclosing molecular mechanisms.

## 2 EXPERIMENTAL SECTION

### 2.1 Bacterial strain

Bacterial strain of *L. paracasei* subsp. *paracasei* M5L (*L. paracasei* M5L) was isolated from kumiss which is a kind of conventional household food in Sinkiang. *Lactobacillus paracasei* M5L was identified by its conservative and polymorphous 16S rDNA. The broth of de Man, Rogosa, and Sharpe (MRS) was used to culture the bacteria at 37°C and stored at 4°C. After two‐time subculture, a 10‐fold serial dilution was plated on MRS medium to determine the colony‐forming units (CFU/ml) of the strain.

### 2.2 Cell line

HT‐29, a human colonic cancer cell line, was provided by the Cancer Institute of the Chinese Academy of Medical Science. RPMI‐1640 medium (Thermo Scientific HyClone) was used to culture the cells, which is supplemented with 10% fetal bovine serum (Thermo Scientific HyClone), penicillin (100 IU/ml), and streptomycin (100 lg/ml), in a 5% carbon dioxide incubator with constant humidity system at 37°C.

### 2.3 Preparation of M5‐EPSs

After incubation at 37°C for 36 hr to denature enzymes, the *L. paracasei*M5L cultures were heated for 15 min at 100°C and then cooled at room temperature. Lactobacilli were removed by centrifugation (10,000 ×g, 4°C) for 30 min, and the supernatant was retrieved for the extraction of M5‐EPSs. Proteins were removed by adding 10% (w/v) trichloroacetic acid and incubating at 4°C overnight; the supernatant was collected after centrifugation (10,000 ×g, 20 min, 4°C). After removing the protein, the product was precipitated with three volumes of ice‐cold ethanol (95%) overnight at 4°C. Subsequently, M5‐EPSs were collected by centrifugation and then dissolved in deionized water and dialyzed against water (4°C, 48 hr) to remove salts, and pure M5‐EPSs were obtained after lyophilization. For further analysis, we used the phenol/sulfuric acid method to determine the total carbohydrate content (98%), and no protein or other components were found.

### 2.4 Cell viability assay

The viability of HT‐29 cells was investigated by an MTT assay. Cells were seeded in 96‐well culture plates at a density of 1 × 10^5^ cells/ml and incubated for 24 hr. Then, a series of concentrations of M5‐EPSs of 20, 250, 500, 500, and 1,000 μg/ml were added to the media for 24, 48, or 72 hr. MTT solution (0.5 mg/ml in media) was added to each well, and then, the plates were incubated for 4 hr at 37°C. After washing, the formazan dye precipitates, which were proportional to the number of live cells, were dissolved in 150 μL DMSO. The amount of formazan product was measured at 490 nm using an enzyme‐linked immunosorbent assay plate reader (Bio‐Rad‐500, Bio‐Rad Laboratories In).

### 2.5 Microscopy observation

The morphological changes of HT‐29 cells were observed using inverted light microscopy after adding 500 or 1,000 μg/ml M5‐EPSs and incubating for 48 hr. Cells grown in the same manner without M5‐EPSs were used as controls. After M5‐EPSs treatment, HT‐29 cells were examined under a microscope, and all photographs were taken with a digital camera.

### 2.6 Transmission electron microscopy

After treatment with 500 and 1,000 μg/ml M5‐EPSs for 48 hr, HT‐29 cells (1 × 10^6^ cells) were harvested by trypsinization. Then, the cells were washed twice with PBS, fixed in 2.5% glutaraldehyde for 90 min, and post fixed in 1% osmium tetroxide for 30 min at room temperature. After washing with PBS, the cells were gradually dehydrated in the upgrading of ethanol (50, 70, 95, and 100%) and embedded in Epon 812 resins. The blocks were cut into ultrathin sections with a microtome and were then stained with saturated uranyl acetate and lead citrate. Transmission electron microscope was used to examine the ultrastructure of the cells.

### 2.7 Flow cytometry analysis

The effects of M5‐EPSs on HT‐29 cell cycle phase distribution were analyzed by flow cytometry. Briefly, HT‐29 cells were treated with M5‐EPSs at concentrations of 500 and 1,000 μg/ml for 48 hr after culture at a density of 5 × 10^5^ cells/ml in 6‐well plates. The cells were harvested by centrifugation at 10,000 rpm for 5 min, washed with 1 ml ice‐cold PBS and fixed with 70% ethanol at 4°C overnight. After centrifugation, the pellet was washed twice with cold PBS and incubated with a PI solution mixed with 100 μg/ml RNase and 0.2% Triton X‐100 for 30 min at 37°C in the dark. Finally, the DNA content was measured via flow cytometry (Becton Dickinson) in triplicate for each experiment, and the percentage of cells in each phase of the cell cycle was analyzed.

### 2.8 Determination of intracellular ROS in HT‐29 cells

Intracellular levels of ROS were measured by determining the ratio of the molecular probe 2′, 7′‐dichlorofluorescindiacetate (DCFH‐DA) to fluorescent 2′, 7′‐dichlorofluorescin (DCF) according to the protocol of the Reactive Oxygen Species Assay Kit (Nanjing Jiancheng Bioengineering Institute). HT‐29 cells were seeded in a 6‐well plate at a density of 5 × 10^5^ cells/ml. After treatment with M5‐EPSs for 48 hr, the HT‐29 cells were incubated with 10 μmol/L DCFH‐DA in the dark for 20 min at 37°C and then washed twice with cold PBS. Finally, the ROS fluorescence intensity of cells was analyzed by a FACScan (Becton Dickinson) flow cytometer with excitation at 488 nm and emission at 530 nm.

### 2.9 Determination of enzyme activities

Cells were incubated with 500 μg/ml and 1,000 μg/ml M5‐EPSs for 24, 48, and 72 hr and lysed in cell lysis buffer. The supernatant was obtained after centrifugation at 12,000 g and collected for the determination of enzyme activities. The antioxidant activities of superoxide dismutase (SOD), catalase (CAT), and glutathione peroxidase (GSH) were measured by spectrophotometry. The commercial kits used in these assays were supplied by the Nanjing Jiancheng Bioengineering Institute (Nanjing, China).

### 2.10 Real‐time PCR

The effect of M5‐EPSs on the gene expression of SOD, CAT, GSH‐Px, caspase‐3, Bax, and Bcl‐2 was detected by real‐time polymerase chain reaction. After NAC treatment, GRP78 and CHOP expression was also detected. Total RNA from HT‐29 cells was isolated using the EasyPure^TM^RNA Kit (Sigma). Complimentary DNA (cDNA) synthesis from total RNA (2 μg) was performed using random primers and the TransScript^®^ One‐Step gDNA Removal and cDNA Synthesis SuperMix (Applied Biosystems). Target transcripts were detected using quantitative real‐time PCR (qRT‐PCR) (TransStart^®^ Top Green qPCRSuperMix, Applied Biosystems). PCR amplification was performed on an ABI Prism 7,300 Sequence Detection System (Applied Biosystems), and the fluorescence was monitored in real time. All reactions were performed in duplicate, and the data represent the means ± *SEM*s of three independent experiments. To exclude variations arising from different inputs of total mRNA into the reaction, all data were normalized to β‐actin, which acted as an internal housekeeping gene. Data were analyzed using the classical delta‐Ct method. Differences were considered significant if *p* < .05.

### 2.11 Statistical analysis

All experiments were performed in triplicate, and all results were expressed as the mean ± *SEM*. Differences observed were assessed using one‐way analysis of variance (ANOVA). Differences were considered to be statistically significant at a value of *p* < .05 in all of the comparisons.

## 3 RESULTS

### 3.1 M5‐EPSs induce cytotoxicity in a dose‐ and time‐dependent manner

**FIGURE 1:**
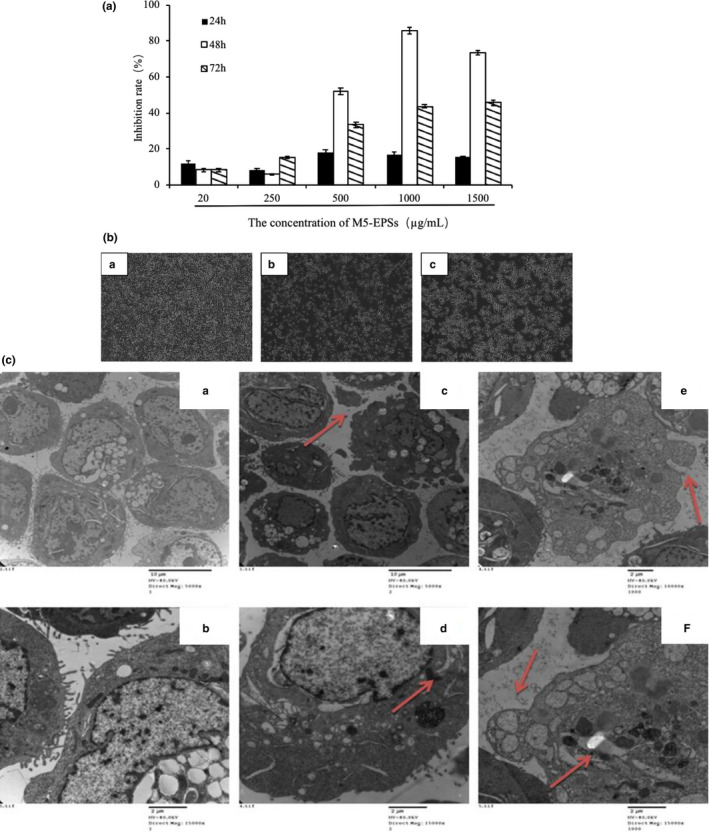
M5‐EPSs effectively induce apoptosis in HT‐29 cells. (a) HT‐29 cells were exposed to various concentrations of M5‐EPSs (0–1,000 μg/ml) for 24, 48, and 72 hr, and M5‐EPSs induced time‐and dose‐dependent cytotoxicity in HT‐29 cells as analyzed by an MTT assay. (b) Numerical and morphological changes of HT‐29 cells as a result of M5‐EPS treatment for 48 hr as assessed with an inverted light microscope. (magnification ×100; A: control group, B: 500 μg/ml M5‐EPSs, C: 1,000 μg/ml M5‐EPSs). (c) Morphology of HT‐29 cells and the endoplasmic reticulum after treatment with 500 and 1,000 μg/ml M5‐EPSs for 48 hr as assessed by TEM. The red arrows represent apoptotic and necrotic cells (A: control group [×5,000], B: control group [×15,000], C: 500 μg/ml M5‐EPSs [×5,000], D: 500 μg/ml M5‐EPSs [×15,000], E: 1,000 μg/ml M5‐EPSs [×5,000], F: 1,000 μg/ml M5‐EPSs [×15,000])

The effect of M5‐EPSs on HT‐29 cell viability was investigated by MTT assay. To determine the antiproliferative effect of time dependence and dose‐dependence on HT‐29 cells, different concentrations of M5‐EPSs (20, 250, 500, 1,000, and 1,500 μg/ml) were administered, and the effect was observed at 24 hr, 48 hr, and 72 hr. As shown in Figure 1‐a, at some lower concentrations, such as 20 μg/ml and 250 μg/ml, M5‐EPSs moderately affected HT‐29 cell viability. At higher concentrations, including 500, 1,000, and 1,500 μg/ml, the inhibition rate increased with increasing concentrations. In addition, HT‐29 cell viability was increased after treatment with M5‐EPSs for 72 hr compared to the effect after 48 hr. Above all, M5‐EPSs markedly reduced cell viability in a dose‐dependent manner from concentration of 20 to 1,000 μg/ml, and 48 hr treatment with M5‐EPSs showed the strongest effect.

### 3.2 Morphological alterations induced by M5‐EPSs

As shown in Figure 1b,c, induction of apoptotic morphology in HT‐29 cells by M5‐EPSs was observed microscopically via inverted light microscopy and TEM. Under an inverted light microscope, the cells showed apoptotic features, the number of cells was reduced greatly (Figure 1b), and remaining cells presented modified extensions in the number, width and length, TEM images showed that the control cells had wide and short cytoplasmic extensions and intact cell surfaces (Figure 1c), while after M5‐EPSs exposure, the abundance of membrane blebs produced in apoptotic cell, as the most prominent characteristic of the treated cells, was occurred. Holes appeared in the membrane or even no membrane of some cells, indicating that they were in very late apoptosis or necrosis. High magnification of the TEM images showed morphological changes in the endoplasmic reticulum (which was deteriorating) in HT‐29 cells after M5‐EPSs treatment. The distribution became uneven, and the endoplasmic reticulum was substantially more swollen in the treatment group than in the control group, with a smooth and unbroken shape, and the changes occurred in a dose‐dependent manner. All of these results support the decreased viability of HT‐29 cells after M5‐EPSs treatment being mediated by the induction of apoptosis.

### 3.3 Cell cycle arrest by M5‐EPSs

**FIGURE 2:**
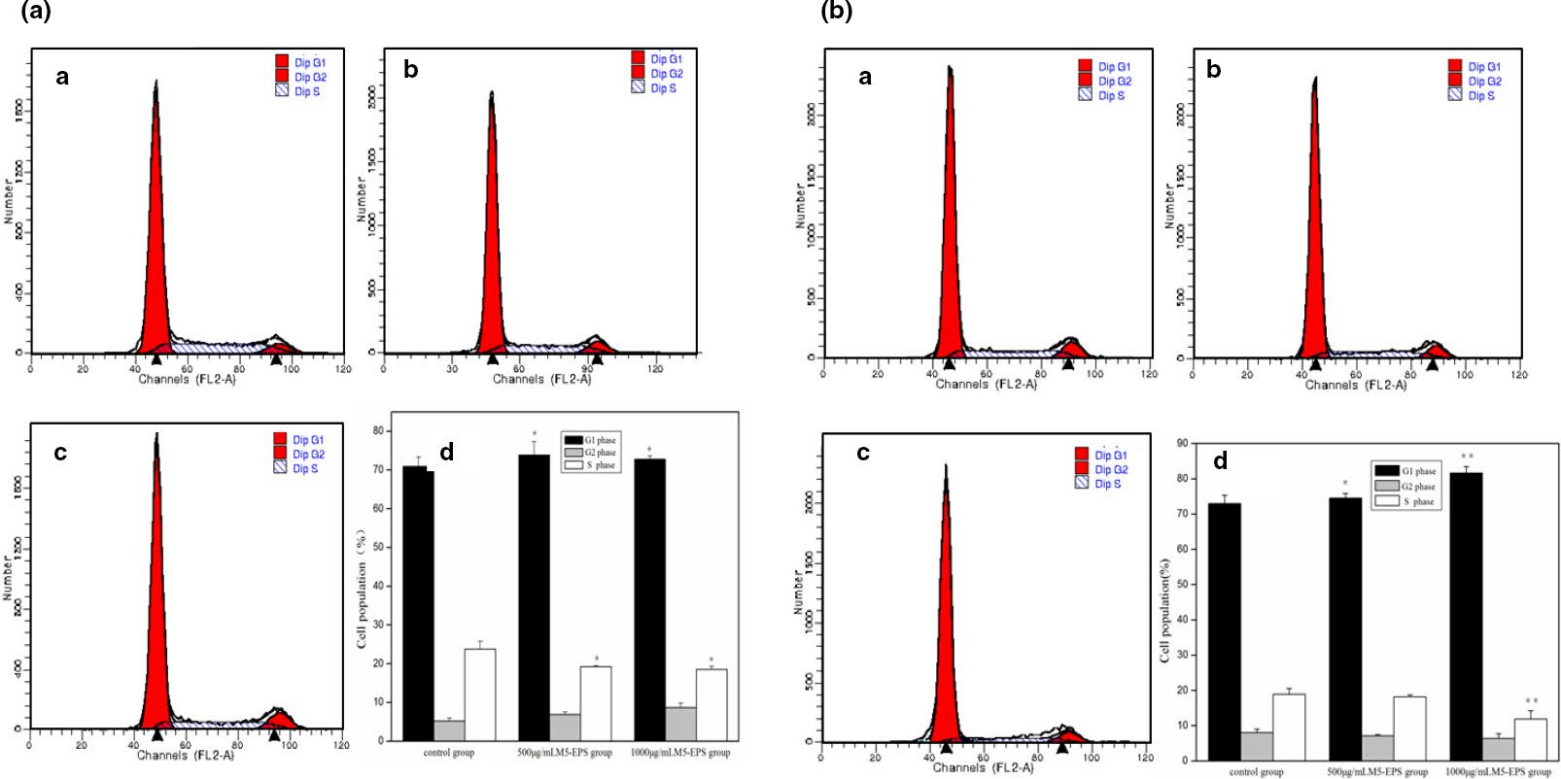
M5‐EPSs induce G0/G1 arrest in HT‐29 cells. (a) HT‐29 cells were incubated with M5‐EPSs for 48 hr and subjected to cell cycle analysis by FCM. (b) HT‐29 cells were incubated with M5‐EPSs for 72 hr and subjected to cell cycle analysis by FCM after incubation with a PI solution (A: control group; B: 500 μg/ml M5‐EPSs; C: 1,000 μg/ml M5‐EPSs; D: cell cycle distribution). Data are expressed as the mean ± *SEM* (*p < .05; **p < .01 compared to control)

The cell cycle includes the G0/G1 phase, S phase, and G2/M phase, which are closely linked to cell apoptosis. As a result of cell cycle arresting, cell growth was suppressed, followed by induction of apoptosis by anticancer substances. To examine the role of M5‐EPSs in the inhibition of HT‐29 cell proliferation, we used flow cytometry to detect the effect of M5‐EPSs on HT‐29 cell cycle distribution. HT‐29 cells were exposed with different concentrations (500 μg/ml and 1,000 μg/ml) of M5‐EPSs for 48 hr or 72 hr. As shown in Figure 2a,b, the percentage of cells in the G1 phase was increased in a dose‐dependent manner, and the percentage of cells in the G0/G1 phase significantly increased (*p* < .05), directly resulting in a remarkably decreased cell number in the S phase (*p* < .05). The above results show that M5‐EPSs may affect HT‐29 cell growth by inducing cell cycle arrest at the G0/G1 phase.

### 3.4 A significant increase in intracellular ROS by M5‐EPSs

**FIGURE 3:**
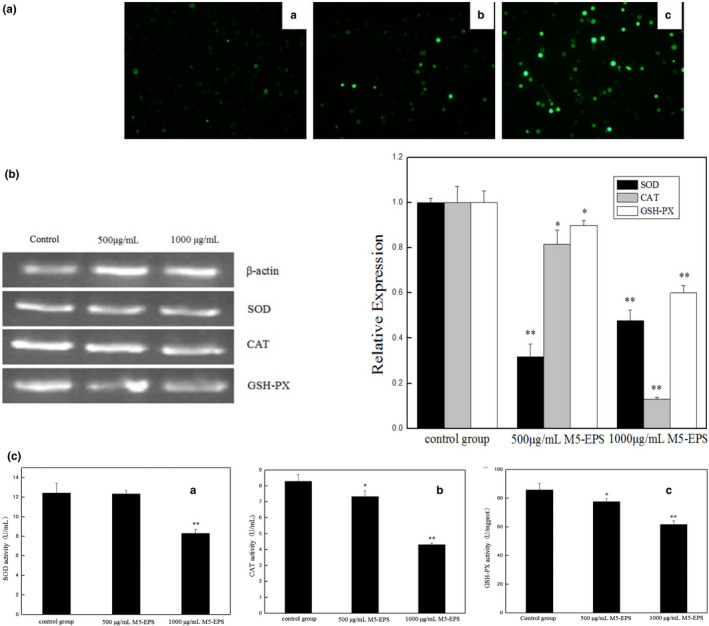
Oxidative stress is induced in HT‐29 cells by M5‐EPSs. HT‐29 cells were treated with M5‐EPSs for 48 hr. (a) Intracellular ROS generation in HT‐29 cells exposed to M5‐EPSs (magnification × 200) (A: control group, B; 500 μg/ml M5‐EPSs, C: 1,000 μg/ml M5‐EPSs). (b, c) Intracellular SOD, CAT, and GSH gene expression and enzyme activities. *p < .05, **p < .01

In living organisms, a moderate increase of ROS is necessary for promoting cells proliferation and differentiation, whereas excessive amounts of ROS cause oxidative damage to cells and is even toxic to cancer cells. To verify whether the apoptosis in HT‐29 cells was mediated by the oxidative stress pathway, we examined the level of ROS in M5‐EPSs‐treated HT‐29 cells. Detection of Intracellular peroxide levels was conducted by flow cytometric analysis using DCFH‐DA as a fluorescent dye. As shown in Figure 3a, M5‐EPSs treatment for 48 hr produced a potent and remarkable increase in ROS in a dose‐dependent manner. These results illustrate that oxidative stress plays an important role in M5‐EPSs‐induced apoptosis.

### 3.5 Effects of M5‐EPSs on CAT, SOD and GSH activity

As the major enzymes with antioxidant activity, antioxidants, including catalase (CAT), superoxide dismutase (SOD) and glutathione peroxidase (GSH), play a significant role in defense against oxidative stress. They are involved in the dismutation reaction of hydrogen peroxide by superoxide radical anions and finally convert hydrogen peroxide to water. As shown in Figure 3c, our results showed that at higher concentrations (1,000 μg/ml), HT‐29 cells treated with M5‐EPSs for 48 hr showed statistically significant depletion of SOD and GSH levels compared to control cells. Similarly, our results also indicated that the level of CAT activity was significantly decreased in a dose‐dependent manner in the different concentrations group. We further examined the levels of genes encoding antioxidant enzymes, such as CAT, SOD and GSH (Figure 3b). After treatment with 500 μg/ml and 1,000 μg/ml of M5‐EPSs for 48 hr, intracellular SOD, CAT and GSH gene expression was significantly reduced in HT‐29 cells compared to the control group. After 500 μg/ml and 1,000 μg/ml M5‐EPSs exposure, the gene expression of SOD was significantly reduced (*p* < .01), but there was no significant difference between the high and low treatment concentrations. With increasing concentrations of M5‐EPSs, CAT and GSH expression were reduced in a dose‐dependent manner, and the 1,000 μg/ml M5‐EPSs treatment significantly (*p* < .01) reduced their activities. These results indicate that downregulation of CAT, SOD and GSH‐Px activity might lead to HT‐29 cell death induced by apoptosis.

### 3.6 M5‐EPSs activate ER stress pathways in HT‐29 Cells

**FIGURE 4:**
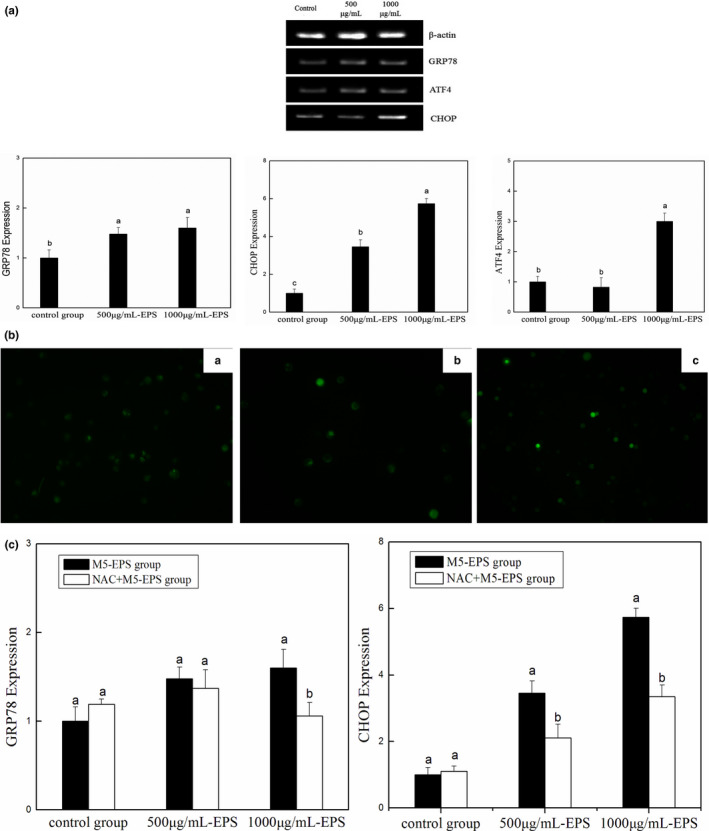
M5‐EPSs induce ER stress in HT‐29 cells. (a) HT‐29 cells were treated with different concentrations of M5‐EPSs for 48 hr. M5‐EPSs effectively increased the expression of GRP78, CHOP and ATF4, indicating that ER stress was involved in the apoptosis triggered by M5‐EPSs. (b) After NAC pretreatment, the level of ROS in HT‐29 cells was suppressed compared with that seen in Figure 3‐a (magnification ×200) (A: control group; B: 500 μg/ml M5‐EPSs; C: 1,000 μg/ml M5‐EPSs). (c) After inhibition of oxidative stress, the expression of GRP78 and CHOP in HT‐29 cells decreased, compared with that in the control group. Different letters indicate significant differences between groups

To investigate the possible mechanism by which M5‐EPSs exert their effects on HT‐29 cells, we further examined the levels of genes encoding GRP78, as well as ATF4, CHOP, which are the typical proteins involved in endoplasmic reticulum stress. Various mechanisms have been suggested to play a role in ER stress‐induced apoptosis. The UPR can reduce ER stress via inducing an initial decrease of general protein synthesis, promoting protein folding by inducing ER chaperones and preventing the accumulation of misfolded proteins. However, if the stress is severe or prolonged, which may transduce distinct death signals during the UPR, and cells will undergo apoptosis. According to the TEM results, after treatment with M5‐EPSs, the endoplasmic reticulum showed a damaged shape. To investigate the role of ER stress in the apoptotic effects of M5‐EPSs treatment in HT‐29 cells, several ER stress‐related molecules were detected. As shown in Figure 4a, M5‐EPSs effectively increased the protein expression of GRP78, CHOP and ATF4, revealing that ER stress participated in the apoptosis triggered by M5‐EPSs in HT‐29 cells. Importantly, the quantitative data showed a significant decrease in GRP78 expression in the 1,000 μg/ml group, and similar trends were also observed in the expression of CHOP and ATF4. All the results suggest that M5‐EPSs can simultaneously induce oxidative stress and ER stress in HT‐29 cells.

### 3.7 Interaction between M5‐EPSs‐mediated modulation of the oxidative stress and ER stress pathway

The above results suggest that M5‐EPSs can stimulate both oxidative stress and endoplasmic reticulum stress in HT‐29 cells. We then added NAC, which is a powerful antioxidant, before 500 μg/ml and 1,000 μg/ml M5‐EPSs treatment. As shown in Figure 4b, NAC treatment decreased the ROS levels in HT‐29 cells, indicating that the oxidant‐induced stress had been suppressed, and GRP78 and CHOP, both important factors related to ER stress, were also significantly decreased (Figure 4c, *p* < .05). These results suggest that M5‐EPSs may cause severe damage to organelles, such as the endoplasmic reticulum of colon cancer HT‐29 cells, by disrupting the oxidative metabolism balance, thus inducing endoplasmic reticulum stress. Once the oxidative stress pathway is inhibited, it affects the transduction of signals related to endoplasmic reticulum stress.

### 3.8 M5‐EPSs‐induced gene expression of caspase‐3, Bax, Bcl‐2 and Bcl‐xl

**FIGURE 5:**
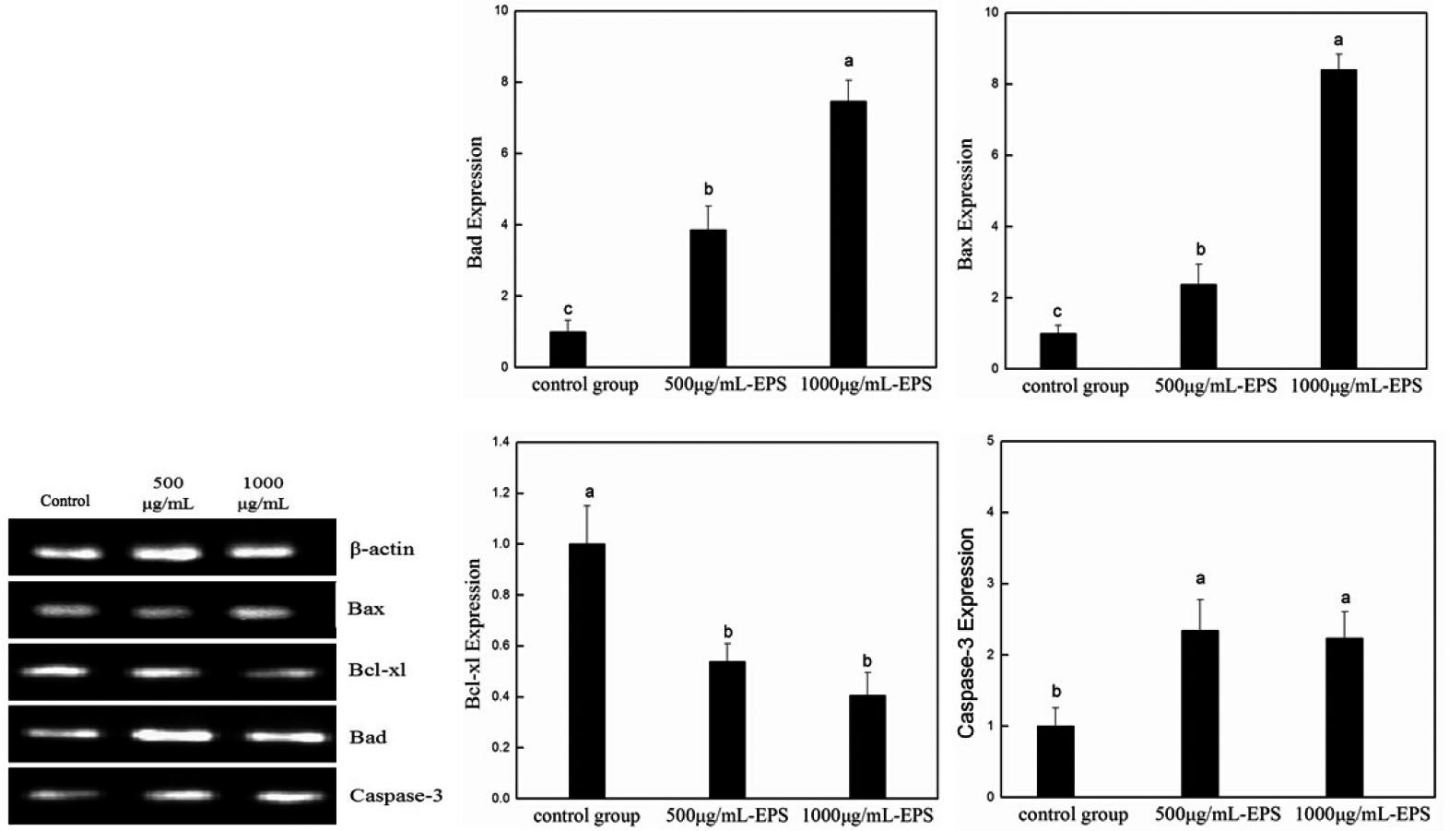
Expression of apoptotic signaling genes of HT‐29 cells (Bad, Bax, Bcl‐xl, and caspase‐3)

The Bcl‐2 protein family and caspases have been implicated in mediating apoptotic signals in response to oxidative stress and ER stress, so we further examined the role of apoptosis‐related proteins by inducing apoptosis using M5‐EPSs in HT‐29 cells. The RT‐PCR data showed that the addition of M5‐EPSs significantly reduced apoptosis in HT‐29 cells. As shown in Figure 5, significant expression of pro‐apoptotic and antiapoptotic Bcl‐2 family proteins, including Bad, Bax, and Bcl‐xl, was observed with RT‐PCR, including a decrease in the antiapoptotic protein Bcl‐xl and an increase in the pro‐apoptotic proteins Bad and Bax. Alternative induction of caspase‐3 was detected in M5‐EPSs‐treated HT‐29 cells compared with the control group. These results indicate that M5‐EPSs‐induced apoptosis is mediated by disruption of the Bcl‐2 family and caspase‐3 in HT‐29 cells.

## 4 DISCUSSION

Tumor cells have a strong ability to proliferate and this ability plays a vital role in the growth and development of tumors (Menyhart et al., 2016). Apoptosis is a regulated and an organized process of programmed cell death (PCD), which plays a crucial role in controlling the development and homeostasis in multicellular organisms (Mondal et al., 2017). Any imbalance between proliferation and apoptosis can lead to carcinogenesis (Kutanzi et al., 2010). Because of their antitumor and immunomodulatory effects and known biological activities, natural polysaccharides have been the topic of much research. Recent evidence has demonstrated that many low‐toxic natural polysaccharides can inhibit tumor cells proliferation and selectively induce apoptosis in various cell lines (Meng et al., 2016).

In this study, M5‐EPSs treatment significantly inhibited HT‐29 cells proliferation and growth in a dose‐dependent manner. After treatment with 1,000 μg/ml EPSs for 48 hr, cell growth was severely inhibited with inhibition rate of 85.85% which was higher than 72 hr treatment. According to the mechanism of MTT assay which we used for measuring cell viability, mitochondrial succinate dehydrogenase activity was actually considered to distinguish live or dead cells. When EPSs attacked HT‐29 cells for longer time of 72 h, heavy ER stress happened as well as mitochondrial injured as alternative pathway since Bac‐xl gene expression decreased. In this case, succinate dehydrogenase would be released and was oxidized by oxide or oxygen molecules after mitochondrial injury. Partial succinate dehydrogenase would not be able to reduce MTT to formazan in purple, as a result, cell viability would be lower in 48 hr rather than 72 h. According to the results, M5‐EPSs strongly inhibited HT‐29 cells proliferation and induced significantly cytotoxic response in a dose‐dependent manner. Apoptotic cells undergo a series of common morphological changes, such as cell shrinkage, nuclear condensation, membrane blebbing, cleavage of chromatin, and formation of pyknotic bodies of condensed chromatin (Henry et al., 2013). Therefore, apoptosis can be recognized by light or electron microscopy through the distinct morphological characteristics of apoptotic cells. In order to determine whether inhibition of proliferation was related to apoptosis, the morphological changes of M5‐EPSs after 48 hr of treatment were observed by inverted light and electron microscopy. The EPSs‐treated cells showed significant changes in morphological characteristics that were consistent with apoptosis, including cell shrinkage, the appearance of small vesicles, cytoplasm condensation, chromatin compaction and nuclear fragmentation. The nuclei appeared to be slightly smaller and had a brighter fluorescence than those in the control, and both the MTT results and the morphological changes proved that M5‐EPSs could induce apoptosis in HT‐29 cells. To confirm the mechanism of inhibition in HT‐29 cells, the effect of M5‐EPSs on cell cycle progression was examined by flow cytometry. The cell cycle is a sequential event and is divided into the G0/G1, S, G2, and M phases. Cell cycle checkpoints, as crucial stages in pathways, are associated with DNA repairs, apoptosis, or differentiation. During cell cycle of G_0_/G_1_ phase, DNA is synthesized, on the other hand, during the G2/M phase, mitotic events occur. In this study, we demonstrated that M5‐EPSs arrested the growth of HT‐29 cells at the G_0_/G_1_ phase and ultimately led to apoptotic cell death in HT‐29 cells.

Both oxidative stress and ER stress have been implicated in promoting cell death, tissue damage, and organ dysfunction. ROS generation could induce the release of the pro‐apoptotic factors and modulate the apoptotic pathway (Song et al., 2014). Antioxidants, such as SOD and CAT, participate in the dismutation reaction of hydrogen peroxide by superoxide radical anions and then convert hydrogen peroxide to water (Ighodaro & Akinloye, 2018). GSH is involved in cell proliferation and promotes tumor metastasis potential. In addition, GSH maintains mitochondrial membrane integrity (Ortega et al., 2008). The imbalance between antioxidants and the ROS level in tumor cells can increase the sensitivity to antitumor agents and cause dysfunction of the endoplasmic reticulum, subsequently inducing cell death by apoptosis. Oxidative stress is usually coupled with ER stress, and the induction of the ER stress response is an event downstream of oxidative stress (Lin et al., 2019). Compared with that in the control group, after 500 μg/ml and 1,000 μg/ml M5‐EPSs treatment, the fluorescence intensity of HT‐29 cells increased in a dose‐dependent manner in the treatment group, indicating that more ROS were generated in the M5‐EPSs treatment group than in the control group. On the other hand, M5‐EPSs reduced the antioxidant activities in HT‐29 cells. SOD, CAT and GSH activity, as measured by mRNA and protein levels, was lower in the treatment group than in the control group. Moreover, we examined ER stress‐related factor expression changes by polymerase chain reaction (PCR). M5‐EPSs significantly increased the expression of the GRP78, CHOP and ATF4 genes, showing that ER stress occurred in HT‐29 cells after treatment with 500 μg/ml and 1,000 μg/ml M5‐EPSs. Moreover, the oxidative stress pathway was inhibited, and endoplasmic reticulum stress was also affected. Recent studies have confirmed that on one hand, ROS is upstream signal factor to trigger ER stress mediated apoptotic pathway which is consistent with our results, on the other hand, ER stress is one of the causes of oxidative stress (Tian et al., 2020; Yang et al., 2019). It was proved that blockage of ROS or ER stress by NAC (N‐acetylcysteine) or 4‐PBA (4‐phenyl butyric acid) induced decrease of ER stress relative factors expression or ROS production respectively. Thus, whether severe ER stress could induce HT‐29 cell oxidative stress by reaction of UPR and mitochondria by M5‐EPSs, would be further uncovered in future.

It is reported that two important pathways are involved in cellular apoptosis: the death‐receptor‐mediated extrinsic pathway and the mitochondria‐dependent intrinsic pathway. Death receptors initiate the extrinsic pathway on the cell surface. The interaction between death receptor and its ligand triggers the formation of a death‐inducing signaling complex, which in turn recruits pro‐caspase 8. Pro‐caspase 8 undergoes autoproteolytic cleavage, forming active caspase 8, an initiator caspase of the extrinsic pathway. The endogenous pathway is initiated when the mitochondrial membrane potential is lost, and then cytochrome c is released from mitochondria to the cytoplasm after the activation of caspase and Bcl‐2 family members. In these two pathways, the activated initiator caspase leads to its own autoactivation, which further activates caspase‐3, the effector caspase. Furthermore, the UPR in the ERS pathway can also activate Bcl‐2 family and caspase proteins (Wong, 2011). In our studies, M5‐EPSs upregulated caspase‐3 expression in HT‐29 cells. Furthermore, M5‐EPSs also upregulated the expression of the pro‐apoptotic protein Bax and downregulated the expression of the antiapoptotic protein Bcl‐2 in HT‐29 cells. These results suggest that M5‐EPSs‐induced apoptosis of HT‐29 cell was associated with both the mitochondrial pathway and the death receptor pathway.

Therefore, EPSs from *L. paracasei* M5L could remarkably induce apoptosis through oxidative stress and the ERS pathway in HT‐29 cells, and they have the potential to be developed into treatments with both health and economic benefits for cancer therapy.

## 5 CONCLUSION

In this study, we extracted M5‐EPSs and cocultured them with HT‐29 cells. After 500 μg/ml and 1,000 μg/ml M5‐EPSs treatment for 48 hr, HT‐29 cells showed obvious apoptosis: cell growth was significantly inhibited, cell size decreased, the structures of the endoplasmic reticulum and mitochondria were destroyed, and apoptotic bodies appeared. Intracellular ROS increased, while antioxidant enzymes decreased, and M5‐EPSs disrupt the balance of the intracellular oxidation system. Moreover, endoplasmic reticulum‐related factors increased significantly, causing the expression of caspase‐3 and Bcl‐2 family proteins. In future study, we will keep investigating the deep mechanism in mRNA level and protein level. In conclusion, M5‐EPSs mediate HT‐29 cell apoptosis through the oxidative stress and endoplasmic reticulum stress pathways.

## CONFLICT OF INTEREST

All authors declare that they have no conflicts of interest.

## Data Availability

The data that support the findings of this study are openly available in [repository name e.g “figshare”] at http://doi.org/10.1002/FSN3.2142[doi], reference number [reference number].
